# Characterization of miR-200 family members as blood biomarkers for human and laying hen ovarian cancer

**DOI:** 10.1038/s41598-020-77068-0

**Published:** 2020-11-18

**Authors:** Pui-Wah Choi, Abbas Bahrampour, Shu-Kay Ng, Sze Kei Liu, Wei Qiu, Fang Xie, Winston Patrick Kuo, Joseph Kwong, Karen H. Hales, Dale B. Hales, Kwong-Kwok Wong, Errol R. Norwitz, Chun Kin Chow, Ross S. Berkowitz, Shu-Wing Ng

**Affiliations:** 1 Department of Obstetrics/Gynecology and Reproductive Biology, Brigham and Women’s Hospital, Harvard Medical School, Boston, MA 02115 USA; 2 MedTimes Molecular Laboratory Limited, Medtimes Medical Group Limited, Unit B, 7/F Roxy Industrial Centre, Kwai Chung, Hong Kong China; 3 WomenX Biotech Limited, Kowloon, Hong Kong China; 4 School of Medicine and Menzies Health Institute Queensland, Griffith University, Nathan, QLD 4111 Australia; 5 Faculty of Health, Kerman University of Medical Sciences, Kerman, Iran; 6 CloudHealth Genomics Limited, Shanghai, China; 7 Department of Obstetrics and Gynecology, Prince of Wales Hospital, The Chinese University of Hong Kong, Shatin, Hong Kong China; 8 Department of Obstetrics/Gynecology, Southern Illinois School of Medicine, Carbondale, IL 62901 USA; 9 Department of Physiology, Biochemistry and Molecular Biology, Southern Illinois School of Medicine, Carbondale, IL 62901 USA; 10 Department of Gynecologic Oncology and Reproductive Medicine, The University of Texas MD Anderson Cancer Center, Houston, TX USA; 11 Newton-Wellesley Hospital, Boston, MA 02462 USA; 12 Present Address: Tufts Medical Center, Tufts University School of Medicine, 800 Washington Street, Boston, MA 02111 USA

**Keywords:** Gynaecological cancer, Biomarkers

## Abstract

MicroRNA-200 (miR-200) family is highly expressed in ovarian cancer. We evaluated the levels of family members relative to the internal control miR-103a in ovarian cancer and control blood specimens collected from American and Hong Kong Chinese institutions, as well as from a laying hen spontaneous ovarian cancer model. The levels of miR-200a, miR-200b and miR-200c were significantly elevated in all human cancer versus all control blood samples. Further analyses showed significantly higher miR-200 levels in Chinese control (except miR-429) and cancer (except miR-200a and miR141) samples than their respective American counterparts. Subtype-specific analysis showed that miR-200b had an overall elevated level in serous cancer compared with controls, whereas miR-429 was significantly elevated in clear cell and endometrioid cancer versus controls. MiR-429 was also significantly elevated in cancer versus control in laying hen plasma samples, consistent with the fact that endometrioid tumor is the prevalent type in this species. A neural network model consisting of miR-200a/200b/429/141 showed an area under the curve (AUC) value of 0.904 for American ovarian cancer prediction, whereas a model consisting of miR-200b/200c/429/141 showed an AUC value of 0.901 for Chinese women. Hence, miR-200 is informative as blood biomarkers for both human and laying hen ovarian cancer.

## Introduction

According to a report published in 2018 by World Ovarian Cancer Coalition, the worldwide number of ovarian cancer cases by 2035 are predicted to increase to 371,000 per year, and deaths will increase by 67% to 254,000^1^. In developed countries, ovarian cancer remains as the deadliest gynecologic malignancy^2,3^. About 20% of women with epithelial ovarian cancer (EOC) are diagnosed at the early stage and the 5-year survival rates can be as high as 90%. In contrast, the 5-year survival rate is around 17–39% when the disease is diagnosed at the advanced stages^4^. Among all the biomarkers, cancer antigen-125 (CA-125) has been the biomarker of choice for managing ovarian cancer patients. However, serum CA125 level has low sensitivity for early diagnosis and the level is affected by conditions such as menstruation, pelvic inflammations, endometriosis, pregnancy, smoking, and intake of caffeine^5,6^. Other biomarkers including glycoprotein human epididymis protein 4, transthyretin, apolipoprotein A1, beta2-microglobulin, and transferrin showed unsatisfactory predictive value^5,7,8^. Therefore, novel biomarkers with high sensitivity and specificity are urgently needed for ovarian cancer diagnosis.

Biomarkers are more informative and may be more specific when their expression alterations are directly caused by genetic and molecular mechanisms underlying the pathophysiological development of the diseases. Mechanism-based biomarkers have utility in the identification of the origin of the disease, differentiation of disease types, and the application of more effective therapies^9,10^. MicroRNAs (miRNAs) play an active role in physio- and pathological processes and can reflect physiological alterations more directly^11^. Indeed, the microRNA profiles in blood exhibited a strong correlation with that in the tumor^12^. In addition, miRNAs are generally more stable than mRNAs in blood^11^ and are good candidates for mechanism-based biomarkers.

MicroRNA-200 (miR-200) family is a master mesenchymal–epithelial transition regulator controlling the expression of epithelial marker E-cadherin^13^. Previously we have shown that the expression of miR-200 and E-cadherin is elevated in human ovarian cancer^14,15^, as well as in laying hen spontaneous ovarian cancer model^16^, the only non-primate animal that develops ovarian adenocarcinoma in nature. Human miR-200 family consists of five members (miR-200a, miR-200b, miR-200c, miR-429, and miR-141) scattered in two separate chromosomal clusters^17^. Interestingly, only the miR-200a, miR-200b, and miR-429 cluster is present in the chicken genome. We have completed a functional study of miR-200, demonstrating that this miRNA family governs inclusion cyst formation in endosalpingiosis, a benign lesion in association with serous ovarian neoplasm, and collective movement involved in ovarian cancer spread^18^. Intriguingly, ectopic expression of miR-200 in normal human ovarian surface epithelial cells induced upregulation of steroid hormone pathway genes^18^, which, in conjunction with frequent ovulation of the laying hens, are consistent with the incessant ovulation hypothesis for ovarian cancer development^19^, and highlight the critical role miR-200 plays in ovarian carcinogenesis^20^. Taken together, miR-200 can be an excellent mechanism-based biomarker for ovarian cancer diagnosis and prognosis.

There have been multiple studies that investigated miR-200 in various diseases such as endometriosis^21^, renal fibrosis^22^, pleural effusion^23^ and colon cancer^24^. There were also a few studies that evaluate miR-200 as a diagnostic or prognostic marker for ovarian cancer (Table S1). Taylor et al. used a microarray platform to compare the miRNA profiles of tumor-derived EpCAM^+^ exosomes isolated from the sera of American women and found that the levels of miR-141, miR-200a, miR-200c, miR-200b, miR-203, miR-205 were elevated in patients with serous papillary adenocarcinoma compared to that isolated from the benign and healthy samples^12^. The second study conducted by Kan et al. in Australia found that miR200a, miR200b and miR200c were highly expressed in sera derived from serous cancer patients when compared to healthy control, with miR-200c the most significantly different between the two groups^25^. In that study, they selected miR-103 from four tested small RNAs as the internal control based on its detectability in sera and no significant differences between the case and control groups. The third study by Zheng et al. was conducted in China and they found nine miRNAs including miR-200a and miR-141 that were detected at higher levels in the plasma from the ovarian cases than in the healthy controls^26^. The fourth study by Zuberi et al. was conducted in India. The levels of miR200a, miR200b and miR200c were revealed higher in the 70 EOC sera samples than 70 sera sample from the healthy controls. The expression of miR-200a was found to be significantly up-regulated in the mucinous and serous subtype, while the level of miR200a and miR-200c were positively correlated to the stages of EOC and metastasis^27^. The fifth study by Gao et al. was conducted in China. MiR-200c and miR-141 were found significantly elevated in the EOC patient sera when compared to healthy controls^7^. Racial disparity, differences in the samples size, forms of blood, methods of blood collection and detection are factors that could contribute to the results found in the above studies.

In this study, we compared the levels of all five miR-200 family members in the blood specimens from patients with EOC and the healthy controls collected in the USA, Hong Kong and China. We also evaluated if there are racial disparities in the blood miR-200 levels, as racial differences in microRNA expression were frequently reported for various diseases^28–30^. As laying hen ovarian cancers express high levels of miR-200 similar to human ovarian cancers^15,16,18^, the miR-200 levels in the plasma samples collected from the healthy and cancerous laying hens were also compared to those in the human blood specimens.

## Results

### Elevated levels of miR-200 members in human ovarian cancer blood specimens and ethnic group difference analysis

Peripheral blood samples were collected initially from a total of 230 female subjects including 112 healthy controls with a median age of 55 and 118 ovarian cancer patients with median age of 57 (Table 1). Clinical characteristics of the ovarian cancer patients are presented in Table S2. The samples were contributed by two US institutions and three sources in Hong Kong/China. All blood samples were processed and reverse transcription-quantitative real-time polymerase chain reactions (RT-qPCR) were performed as described in Materials and Methods. To choose the endogenous normalizer for the miRNA expression, we tested two miRNAs recommended by the manufacturer and, similar to Ken et al.^25^, chose the same miR-103a-3p as the endogenous reference miRNA to normalize all our RT-qPCR results, as it did not show significant variations in a preliminary test of 40 normal and cancer plasma samples (Figure S1). We noted that serum and whole blood samples were provided by Hong Kong MedTimes and CloudHealth Genomics Ltd, respectively, while the blood samples provided by other institutions were plasmas. We compared the miRNA levels in 10 MedTimes specimens, which were available in all three forms. Statistical analysis of qPCR data showed that the miRNA levels in most serum and plasma specimens corroborated each other, whereas the whole blood samples were significantly different from the other two forms (Figure S2). Glinge et al. have shown that long-term storage of whole blood samples might change miRNA stability^31^. We therefore excluded the 16 CloudHealth whole blood samples from final analysis. miR-103a-3p normalized − ΔCt values^32^ were used to represent miR-200 levels for all the plasma samples as well as the 60 serum samples from Hong Kong MedTimes. In total there were 96 normal and 118 cancer human specimens for the final analysis.

**Table 1:** Sample distribution in this study.

	Sample type	Normal	Cancer	Subtype distribution of cancer			
				Serous	Endometrioid	Clear cell	Mucinous
**Human samples**							
**American institutions**							
Brigham and Women's Hospital	Plasma	10	24	22	2	0	0
MD Anderson Cancer Center	Plasma	25	25	25	0	0	0
Total		35	49	47	2	0	0
**Chinese institutions**							
Chinese University of Hong Kong	Plasma	1	69	21	16	17	15
CloudHealth genomics	Whole blood	16	0				
Hong Kong MedTimes	50 Serum and 10 (Serum + Plasma + Whole blood)	60	0				
Total		77	69	21	16	17	15
**All human samples**		112	118	68	18	17	15
**Laying hen samples**	Plasma	11	15				

The initial analysis of all 214 human samples using linear regression models with adjustment for institutional effects showed that miR-200a, miR-200b, and miR-200c in cancer samples have significantly higher levels than in normal samples (Table 2 and Figure S3). However, further analysis of the data showed that there were significant differences between American and Chinese samples. Within the 84 American samples, cancer samples have significantly higher miR-200a, miR-200b, miR-200c, and miR-141 levels (in average 18.6-fold, 21.7-fold, 23.2-fold, and 22.5-fold, respectively) than normal samples, whereas within the 130 Chinese samples, only miR-200b and miR-429 levels (64.1-fold and 19.9-fold, respectively) were significantly higher than normal samples, and the levels of miR-141 in cancer blood samples were actually significantly lower (0.05-fold) than in normal samples (Table 2). Within the normal samples, four miR-200 family members except miR-429 were significantly higher in Chinese samples than their American counterparts; and within the cancer samples, the miR-200b, miR-200c, and miR-429 levels were significantly higher in Chinese samples than the American samples (Table S3).

**Table 2:** Normalized miR-200 family levels (estimated means of − ΔCt, 95% CI) in (a) All human, (b) American-only, or (c) Chinese-only blood samples.

	Normal	Cancer	Fold-change (cancer/normal)	*P* value^
**(a) All human samples (N = 214)**				
miR-200a	− 21.1 (− 23.5, − 18.8)	− 16.2 (− 18.3, − 14.2)	29.9 (2.3, 390.2)	0.010
miR-200b	− 21.3 (− 23.7, − 18.8)	− 15.5 (− 17.6, − 13.3)	55.3 (3.8, 802.9)	0.003
miR-200c	− 17.5 (− 19.9, − 15.2)	− 12.2 (− 14.3, − 10.1)	40.0 (3.0, 526.8)	0.005
miR-429	− 19.4 (− 23.6, − 15.3)	− 16.5 (− 18.1, − 14.9)	7.4 (0.3, 163.0)	0.203
miR-141	− 15.2 (− 19.3, − 11.1)	− 15.8 (− 17.4, − 14.2)	0.67 (0.03, 14.2)	0.794
**(b) American-only samples (N = 84)**				
miR-200a	− 23.0 (− 26.0, − 20.0)	− 18.8 (− 21.2, − 16.4)	18.6 (1.3, 267.2)	0.032
miR-200b	− 22.0 (− 25.0, − 18.9)	− 17.5 (− 19.8, − 15.2)	21.7 (1.5, 308.0)	0.023
miR-200c	− 20.1 (− 23.2, − 17.1)	− 15.6 (− 17.9, − 13.3)	23.2 (1.6, 332.0)	0.021
miR-429	− 17.7 (− 20.8, − 14.6)	− 18.0 (− 20.4, − 15.6)	0.82 (0.06, 12.2)	0.885
miR-141	− 21.0 (− 24.1, − 17.8)	− 16.5 (− 18.9, − 14.1)	22.5 (1.4, 357.1)	0.028

	Normal	Cancer	Fold-change (cancer/normal)	*P* value^#^
**(c) Chinese-only samples (N = 130)**				
miR-200a	− 16.8 (− 19.0, − 14.7)	− 16.0 (− 18.1, − 14.0)	1.7 (0.2, 13.8)	0.592
miR-200b	− 19.4 (− 21.8, − 17.1)	− 13.4 (− 15.7, − 11.2)	64.1 (7.0, 584.9)	< 0.001
miR-200c	− 12.3 (− 14.5, − 10.1)	− 10.5 (− 12.5, − 8.4)	3.6 (0.5, 29.2)	0.222
miR-429	− 18.0 (− 19.9, − 16.0)	− 13.6 (− 15.4, − 11.8)	19.9 (3.2, 122.9)	0.001
miR-141	− 10.2 (− 12.0, − 8.3)	− 14.5 (− 16.2, − 12.7)	0.05 (0.01, 0.29)	0.001

The normalized miRNA levels are presented as − ΔCt values of RT-qPCR results.

^*P* value using linear regression models with adjustment for institutional effects.

^#^*P* value using linear regression models without adjustment for institutional effects due to confounding with normal and cancer group comparison.

### Correlation analysis of levels of miR-200 family members in human blood samples

To account for the observed racial differences, we compared the correlations of the levels of miR-200 family members in all samples and within different subgroups. When analyzed in all 214 samples, the levels of most miR-200 family members were significantly correlated with each other, except between miR-429 and miR-141 (Table S4). We saw similar correlation pattern in the analysis of all 118 cancer cases, with insignificant correlation between miR-429 and miR-141, and higher positive correlation coefficients among the other members (Table S5). However, the correlations were much weaker except between miR-200c and miR-141 in the analysis within all 96 normal controls, with miR-429 showing either insignificant or negative correlations with the other members (Table S6). When the analysis was performed within the American samples (N = 84), we saw significant correlations between miR-200c and miR-200a and miR-200b, as well as between miR-141 and all miR-200 members except miR-429 (Table S7). The same correlation pattern was also observed in the analysis within American cancer samples (N = 49), with miR-429 not showing any significant correlations with other members (Table S8). Within American normal samples (N = 35), correlations were only observed between miR-200b and miR-200c and miR-141, and negatively with miR-429 (Table S9). When the analysis was performed within the Chinese group (N = 130), all miR-200 family members showed significant correlations with each other except between miR-429 and miR-141 (Table S10), similar to what we observed in the analysis of all samples (Table S4). Like the American cancer counterparts, Chinese cancer samples (N = 69) showed the same pattern as the analysis for all Chinese samples, with even higher significant correlation coefficients (Table S11), suggesting that the correlation pattern observed with all samples was primarily contributed by the elevated levels of miR-200 family in cancer samples. In the analysis within Chinese normal samples (N = 61), only the levels between miR-200a and miR-200b, and the levels between miR-141 and miR-200b and miR-200c showed weak significance (Table S12).

### Elevated levels of miR-429 in human endometrioid and laying hen ovarian cancers

We then proceeded to compare the miR-200 levels across different histological subtypes of ovarian cancer. We chose to analyze only the Chinese samples, which had comparable number of samples derived from different histological subtypes. By Dunnett multi-comparison, we showed that serous and mucinous ovarian cancer blood samples had significantly higher levels of miR-200b (670.5-fold and 267.1-fold, respectively) than normal blood samples; serous cancer blood samples also had significantly higher levels of miR-200c (198.2-fold) than normal; miR-429 levels in clear cell and endometrioid cancer blood samples were significantly higher (64.9-fold and 88.8-fold, respectively) than normal; whereas miR-141 levels in clear cell, endometrioid, and mucinous cancer blood samples were significantly lower (0.01-fold, 0.01-fold, and 0.03-fold, respectively) than normal blood samples (Table 3). Interestingly, when we examined miR-200 levels in the plasma samples from laying hens, only miR-429 showed significantly elevated levels (17.3-fold) in the cancer plasmas compared to control (Table 4 and Figure S4). Correlation analysis showed that chicken miR-200a and miR-200b levels had the highest correlation (Spearman correlation coefficient = 0.817, *P* < 0.001). MiR-429 also showed respectably high correlations with miR-200a (0.535, *P* = 0.005) and miR-200b (0.661, *P* < 0.001) (Table S13). As endometrioid ovarian cancer is the major histological type in the chicken^33^, the laying hen finding is also consistent with the significant increase of miR-429 levels in human endometrioid blood samples.

**Table 3:** Significant miR-200 family levels in human blood samples from different histological types of ovarian cancer versus normal blood samples.

miRNA	Normal	Serous	Clear cell	Endometrioid	Mucinous	*P* value^
miR-200b	− 20.2 (7.7)	− 10.8* (11.3) 670.5* (10.9, 41418.9)	− 17.2 (10.1) 7.9 (0.1, 678.4)	− 14.1 (10.6) 66.7 (0.7, 6356.4)	− 12.1* (7.0) 267.1* (2.5, 28526.2)	0.001
miR-200c	− 12.6 (6.3)	− 5.0* (11.3) 198.2* (4.3, 9146.5)	− 14.2 (7.9) 0.34 (0.01, 21.48)	− 11.0 (8.8) 3.1 (0.04, 211.0)	− 13.3 (10.5) 0.63 (0.01, 48.0)	0.005
miR-429	− 18.2 (5.2)	− 15.1 (7.4) 8.7 (0.3, 272.7)	− 12.2* (9.7) 64.9* (1.6, 2698.6)	− 11.8* (8.6) 88.8* (2.0, 4020.1)	− 15.2 (10.8) 8.4 (0.2, 416.7)	0.011
miR-141	− 10.7 (5.7)	− 9.2 (8.8) 2.9 (0.1, 71.4)	− 17.1* (7.3) 0.01* (0.0004, 0.40)	− 17.1* (8.2) 0.01* (0.0004, 0.41)	− 16.0* (7.3) 0.03* (0.0007, 0.95)	< 0.001

Data are mean (standard deviation) in the first row and mean fold-change relative to normal (95% CI) in the second row.

^*P* value using ANOVA.

Dunnett test relative to Normal (*P value < 0.05).

**Table 4:** Comparison of miR-200 family levels between ovarian cancer and normal laying hen blood samples.

miRNA	Normal	Cancers	*P* value^
miR-200a	− 8.2 (7.4)	− 5.5 (9.5) 6.6 (0.6, 70.3)	0.119
miR-200b	− 5.4 (4.1)	− 1.6 (6.8) 13.2 (0.8, 232.2)	0.078
miR-429	− 8.7 (4.7)	− 4.6 (4.7) 17.3* (1.4, 208.5)	0.025

Data are mean (standard deviation) in the first row and mean fold-change relative to normal (95% CI) in the second row.

^*P* value using Mann–Whitney test.

### Multilayer perceptron neural network models to predict different miR-200 combinations for American and Chinese ovarian cancer patients

**Figure 1:**
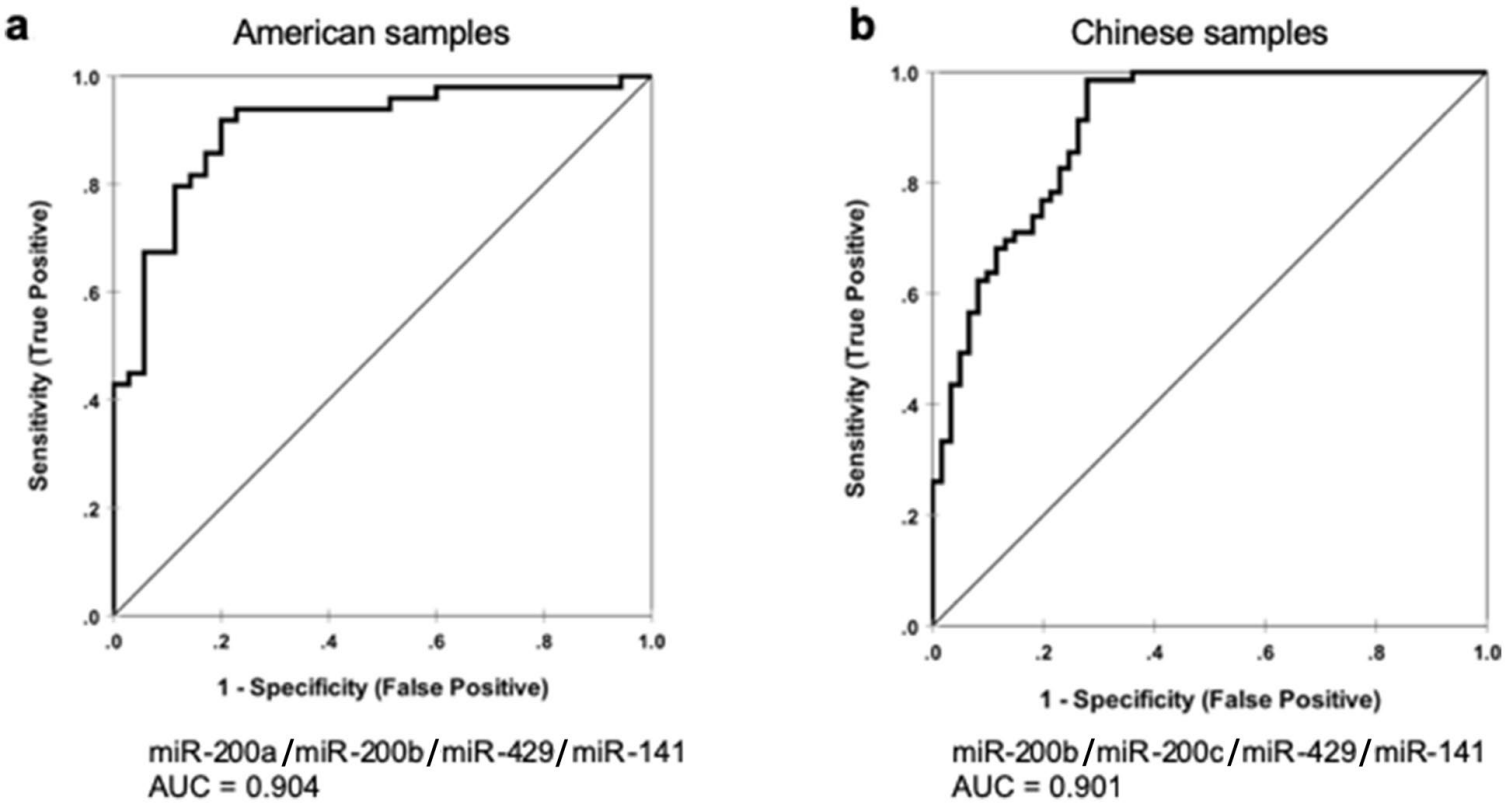
Receiver operating characteristic (ROC) curves for the best multilayer perceptron neural network predictive models to distinguish cancers from non-cancer controls for (**a**) American patients; and (**b**) Chinese patients.

Multilayer perceptron (MLP) neural network predictive models^34^ were used to estimate separately individual and combinations of miR-200 members in differentiating cancer and control blood samples as described in Materials and Methods. The best neural network model consisting of miR-200a/200b/429/141 showed an AUC value of 0.904 for American women in distinguishing between cancer patients and controls, whereas a model consisting of miR-200b/200c/429/141 showed an AUC value of 0.901 for Chinese ovarian cancer prediction (Fig. 1).

## Discussion

We examined levels of miR-200 family members in normal and cancer blood specimens collected from American and Chinese institutions. Statistical analyses have revealed that miR-200a, miR-200b, and miR-200c were significantly elevated in cancer specimens relative to controls in the analysis of all specimens, which are similar to the findings by Taylor et al.^12^, Kan et al.^25^, and Zuberi et al.^27^. Correlation analysis has shown that the levels of these three miR-200 family members were consistently highly correlated with each other than miR-429 and miR-141, with the latter two showing discordant correlation with each other.

Further analysis of the American plasma samples showed that all miR-200 family members except miR-429 were elevated in cancer relative to control plasma samples (Table 2), which is also consistent with the finding of another American miRNA biomarker discovery study using tumor serum-derived exosomes reported by Taylor et al.^12^, further confirming that plasma and serum specimens have similar miRNA levels and consistent elevation of these four miR-200 family members in American ovarian cancer patients. Similar findings of elevated miR-200a, miR-200b, miR-200c levels in cancer serum samples were also found in the Australian study^25^ and the Indian study^27^. However, our analysis of Chinese specimens revealed that Chinese cancer blood samples had significantly higher levels of miR-200b and miR-429 and lower miR-141 levels than in normal samples (Table 2), which is contradictory to two Chinese studies, which showed either increased levels of plasma miR-200a and miR-141^26^, or serum miR-200c and miR-141^7^ in cancer than in normal blood samples. The differences might be due to the use of different endogenous normalizer, the form of the blood samples, and the widely variable miR-141 levels observed in our study.

Correlation analysis of the miR-200 family levels show that the levels of miR-200 members were highly correlated with each other in the cancer samples with the exception of miR-429 in American cancer samples (Tables S5, S8, and S11), which also strongly represented the patterns observed in the analysis of combined cancer and normal samples (Tables S4, S7, and S10) than the normal samples (Tables S6, S9, and S12). This observation implies that normal blood samples usually have low and variable miR-200 levels, whereas cancer samples have consistently elevated levels of miR-200 members and dominate the patterns revealed from combined samples.

The significant positive association of clear cell and endometrioid ovarian cancers with miR-429 and negative association with miR-141 in our histological subtype analysis (Table 3) may account for the differences between American and Chinese cancer samples (Table 2) and differences in correlation analysis (Tables S8 and S11). Since our American cancer samples are primarily serous cancer type and have higher levels of all miR-200 family members except miR-429, and our Chinese samples have significant contribution from clear cell and endometrioid cancer samples, hence we find significant positive contribution by miR-429 in Chinese cancer cases (Table 2 and Table S11), but not in the American cases (Table 2 and Table S8). Future studies with more balanced numbers of histological subtypes in both ethnic groups will confirm the interesting finding of racial disparity of miR-200 blood levels, as there are reports about racial differences in microRNA expression in other diseases^28–30^. More importantly, the human endometrioid cancer-specific miR-429 association is also observed in the cancer plasma samples of laying hen (Table 4), which is prevalently associated with endometrioid ovarian tumors. This finding not only supports the incessant ovulation hypothesis, but also highlights the importance of miR-200-mediated ovarian carcinogenesis in both species^15,16,18^.

In conclusion, we show that miR-200 family is a highly relevant and informative mechanistic biomarker for ovarian cancer, demonstrating outstanding AUC values in our neural network modeling. Our study also shows racial disparity of miR-200 level in the blood, which might contribute to different miR-200 combinations identified in our neural network models for American and Chinese populations (Fig. 1). More importantly, miR-200 family members provide histological subtype-dependent information in both human and laying hen blood samples and have important implication in ovarian carcinogenesis. Longitudinal laying hen blood samples collected before and after tumor development may be useful for ovarian cancer biomarker discovery.

## Materials and methods

### Human and laying hen blood specimens

All methods were carried out in accordance with relevant guidelines and regulations for both humans and animals. Informed consent was obtained from all human subjects. All patient-derived biologic specimens were collected and archived under protocols approved by the respective Human Subjects Committee of the institutions, namely, Human Subjects Committee of the Brigham and Women's Hospital, Institutional Review Board of The MD Anderson Office of Human Subjects Protection, The Joint CUHK-NTEC Clinical Research Ethics Committee, Medtimes Medical Group Ethics Review Board, and CloudHealth Medical Group Clinical Research Review Board. For the American samples, 34 banked plasma samples (10 normals and 24 cancers) at Brigham and Women’s Hospital (BWH) were selected through a Biobank Portal query tool and requested from Partners HealthCare Biobank (https://personalizedmedicine.partners.org/Biobank/Default. aspx). 50 plasma specimens (25 normals and 25 cancers) were from MD Anderson Cancer Center, obtained from the Blood Specimen Research Resource (BSRR) (https://www.mdanderson.org/research/departments-labs-institutes/programs-centers/center-for-translational-and-public-health-genomics/resources.html). A search of databases showed that not any single specimen was from Asian patients. For the Chinese samples, 70 plasma samples were collected at the Department of Obstetrics and Gynecology, Prince of Wales Hospital, Chinese University of Hong Kong. 16 whole blood of healthy Chinese female individuals with no known diseases were collected at CloudHealth Genomics Limited. In addition, 60 serum samples of healthy Chinese females were collected at MedTimes Molecular Laboratory Limited. Of the 60 samples, 10 samples had accompanied plasma and whole blood for comparison. Peripheral blood samples were collected in color-coded Vacutainer tubes (BD Biosciences, San Jose, CA) using routine phlebotomy techniques and fractionated into multiple aliquots and stored at − 80 °C.

Single-comb White Leghorn hens were maintained as previously described^35,36^, with review and approval of the Institutional Animal Care and Use Committees at the University of Illinois at Urbana-Champaign and Southern Illinois University at Carbondale. The plasma samples tested were from age-matched laying hens fed with standard commercial layer feed.

### Handling of blood specimens

All the blood specimens were sent to Brigham and Women’s Hospital in dry ice and processed by the first author except for the whole blood and serum samples from Chinese healthy women. The whole blood samples from CloudHealth Genomics were processed by the fifth author in Shanghai because of the strict policy for exporting biological samples, while the serum samples were processed in MedTimes Molecular Laboratory by the first author. Nevertheless, the same RNA extraction and RT-qPCR procedure were followed for all plasma, sera and whole blood specimens.

### RNA extraction and reverse transcription

All samples were centrifuged at 2000 rpm for 5 min to remove remaining blood cells. One hundred thirty microliters of human blood sample or 50 μl of chicken plasmas were transferred to a 1.5 ml tube and top up to 200 μl with RNase free water. RNA extraction was performed according to miRCURY™ RNA Isolation Kit-Biofluids Instruction manual v1.7 (#300112 and #300113, Exiqon, Woburn, MA). Briefly, lysis buffer was added to the samples, followed by protein precipitation solution. After centrifugation, supernatant was transferred to a new collection tube. RNA was precipitated with glycerol-isopropanol mix. The microRNAs were captured by microRNA Mini Spin Column and eluted with RNase free water. Samples were stored at − 80 °C until reverse transcription was performed.

Reverse transcription was performed according to miRCURY LNA™ Universal RT microRNA PCR Instruction manual v6.1 (#203301, 203351, Exiqon, Woburn, MA). Spike-in RNA control (UniSp6) was included in the reverse transcription reaction to reveal the efficiency of reverse transcription and the subsequent PCR reactions. Normalization was done by subtracting the Ct value obtained with the spike-in RNA (UniSp6). Two microliters of RNA template and 8 μl of working master mix solution containing reaction buffer, enzyme for reverse transcription and synthetic RNA spike-in control were mixed together and incubated at 42 °C for 60 min, followed by 5 min at 95 °C.

### Quantitative PCR reactions

We employed Exiqon (Woburn, MA) miRCURY LNA™ Universal RT microRNA PCR primer sets because of the exceptional sensitivity and specificity and low background. cDNA was diluted 40-fold with nuclease-free water before PCR amplification. cDNA from human samples were mixed with PCR master mix containing primers for different miR-200 members, UniSp6, or the endogenous reference miRNAs. cDNA from chicken samples were mixed with PCR master mix containing compatible human miR-200a, miR-103a-3p, or chicken (gga)-miR-200b, miR-429, or UniSp6. All PCR reactions were performed in a StepOne Plus or a 7500 real-time PCR instrument with 60 amplification cycles (95 °C for 10 s, followed by 60 °C for 1 min). Melt curve analysis was done to reveal primer dimer formation or non-specific binding. Ct value of each reaction was obtained with threshold set to 5000. Ct values larger than 60 were counted as 60. All reactions were performed in duplicates and the average Ct values were used to compute normalized miRNA level for each human and chicken sample.

### Statistical analysis

Comparative − ΔCt (negative delta Ct) method was used to calculate normalized miRNA level for each sample^32^. Normalized delta Ct value was calculated as [Ct(target) − Ct(UniSp6) − Ct(103a)]. Mean levels of miR-200 family members between groups were compared using independent-sample *t* test and regression analysis. Mean fold-changes of miR-200 expression in various histological types of cancer relative to normal samples were calculated using the 2^−ΔΔCt^ method. Levels of miR-200 members in the laying hen groups were compared using Mann–Whitney test due to small sample size. Histological subtype-specific levels of miR-200 members were analyzed using ANOVA with Dunnett post-hoc test comparing each subtype versus normal samples. Pearson correlation coefficients were calculated for correlation analysis in human samples and Spearman correlation coefficients for the laying hen samples. Multilayer perceptron (MLP) neural network predictive models^34^, separately for each marker and for a combination of markers, were estimated to differentiate between cancer and control blood samples. Sensitivity analyses were conducted to compute the importance of each marker in determining the neural network predictive models. Validation of predictive models was performed by randomly dividing the samples into a training data set and a test data set in a ratio of 7:3. The receiver operating characteristic (ROC) curve for each predictive model was obtained and the corresponding area under curve (AUC) was reported. Statistical analyses were performed using IBM SPSS-25 (IBM, Chicago, IL). For all statistical comparisons, a level of *P* < 0.05 was accepted as statistically significant.

## Supplementary information

Supplementary Information 1.

## Data Availability

All data generated of the current study are available from the corresponding authors on reasonable request.
