# Supplementary material for: Characterization of miR-200 family members as blood biomarkers for human and laying hen ovarian cancer
Source: Sci Rep. 2020 Nov 18;10:20071. doi: 10.1038/s41598-020-77068-0 (PMC7674435; doi:10.1038/s41598-020-77068-0)
Supplement: Supplementary file 1 — Supplementary Information 1. [file 41598_2020_77068_MOESM1_ESM.pdf]

Supplementary Information for

Characterization of miR-200 family members as blood biomarkers for human and laying hen ovarian cancer

Pui-Wah Choi, Abbas Bahrampour, Shu-Kay Ng, Sze Kei Liu, Wei Qiu, Fang Xie, Winston Patrick Kuo, Joseph Kwong, Karen H. Hales, Dale B. Hales, Kwong-Kwok Wong, Errol R. Norwitz, Chun Kin Chow, Ross S. Berkowitz and Shu-Wing Ng

4 Figures and 13 Tables

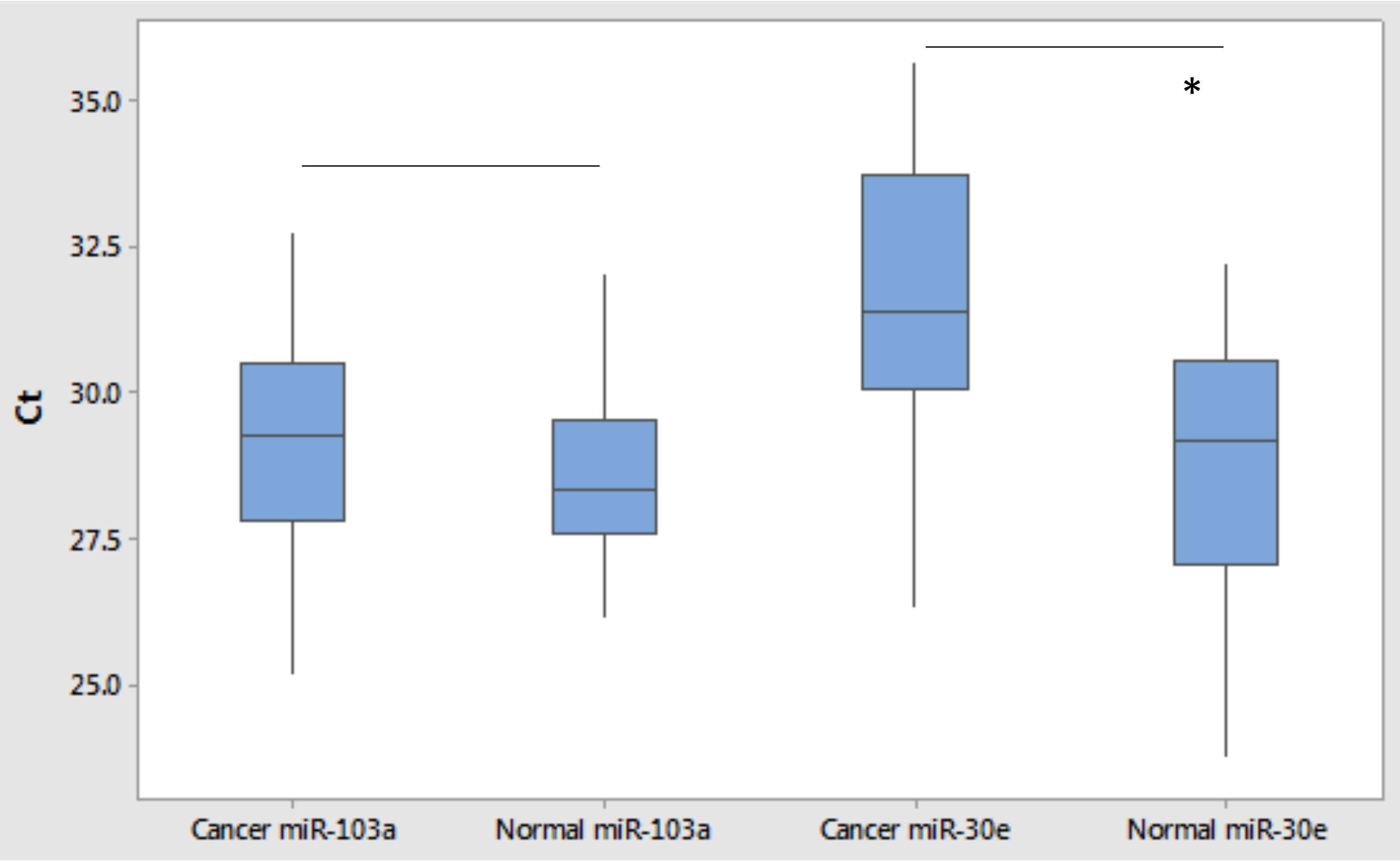

Figure S1. Evaluation of endogenous control for miR-200 normalization.

Boxplots showing the Ct values of miR-103a and miR-30e in 20 normal women and 20 ovarian cancer patients. * $P < 0.05$.

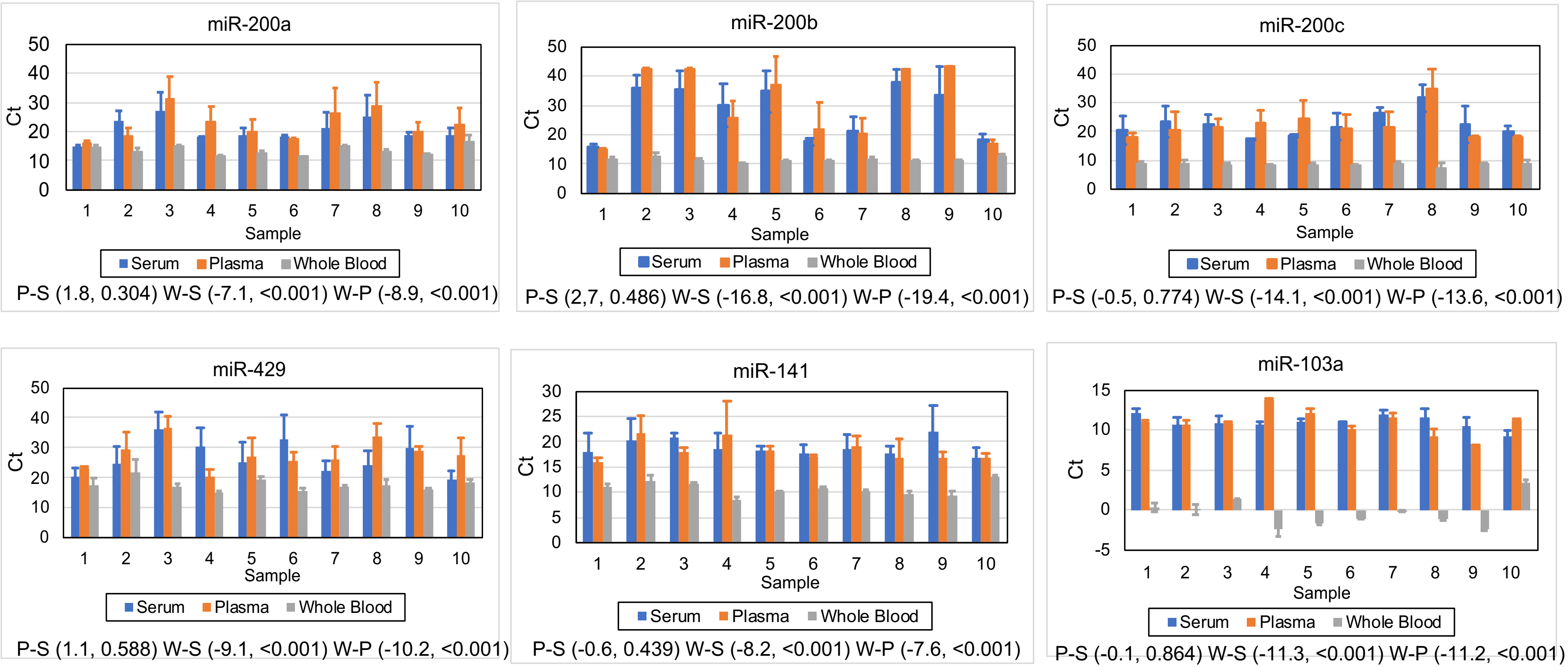

Figure S2. Levels of miRNAs in different forms of blood samples.
The levels of miRNA-200 family and miR-103a presented as Ct values were plotted for the plasma, serum, and whole blood specimens of ten individuals.
Below each graph, the results of statistical analysis of triplicate qPCR data using linear mixed modeling on differences between plasma (P), serum (S) and whole blood (W) samples are presented. The first numbers show the estimated mean differences, whereas the second numbers represent the P-values.

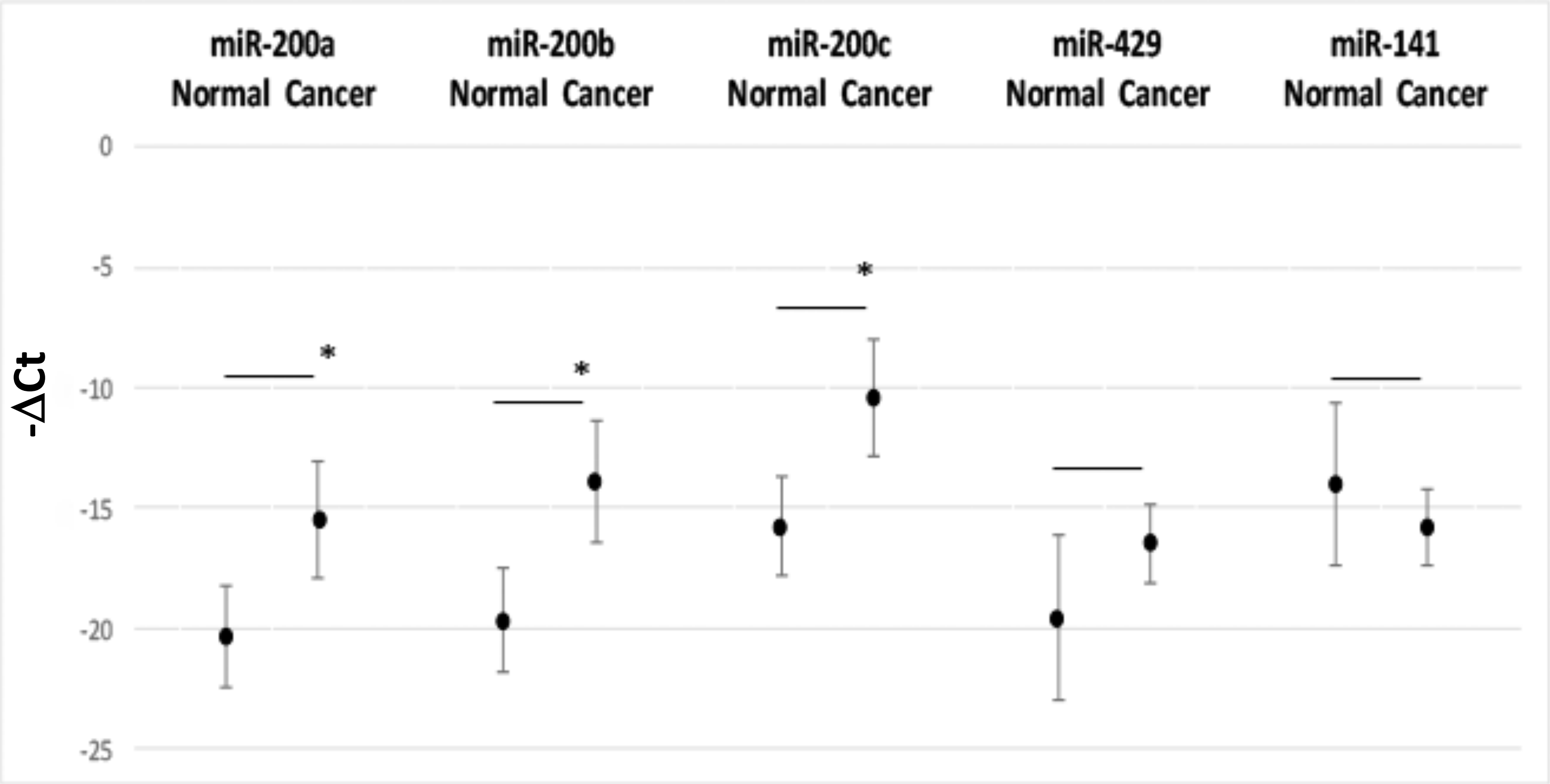

Figure S3. Estimated mean (95% CI) levels of each miR-200 family member were plotted between Normal and Cancer human blood samples.
* *P* < 0.05.

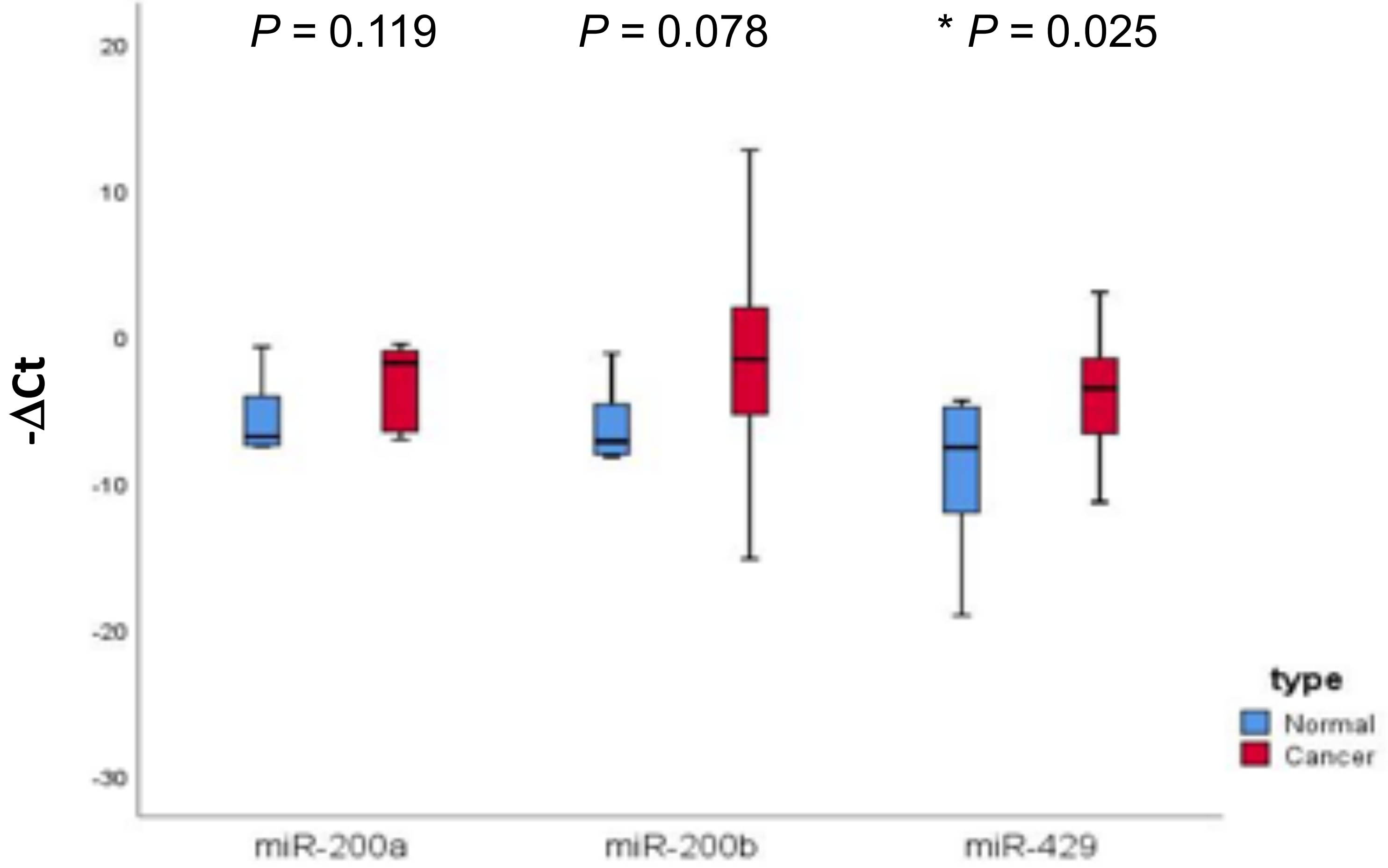

Figure S4. Boxplots showing the levels of miR-200 family members in Normal and Cancer laying hen plasmas. * indicates significance.

Table S1. Characteristics of the previous studies that evaluated the miR-200 family as an ovarian cancer biomarker

Reference	Year	Country	Method	Members of miR-200	Source	Histologic Subtypes	Sample size	Endogenous Normalizer
Taylor et al. (12)	2008	USA	miRNA profiling using microarrays with probes for 467 human mature miRNAs	200a, 200b, 200c, 141	Exosome in Serum	Serous Papillary Adenocarcinoma of the ovary	50 EOC (10 Stage 1, 10 Stage 2, 20 Stage3, 10 Stage 4),<br>10 Benign, 10 Healthy	Microarray Control miRNAs (Ambion)
Kan et al. (25)	2012	Australia	RT-qPCR- TaqMan miRNA assays	200a, 200b, 200c	Serum	Serous epithelial ovarian cancer	28 EOC, 28 Healthy	Tested: miR-103a, miR-92a, miR-638, RNU48.<br>Selected: miR-103a
Zheng et al. (26)	2013	China	RT-qPCR- TaqMan miRNA assays	200a, 141	Plasma	Serous, Mucinous, Clear cell, Endometrioid	76 EOC, 30 Healthy	No endogenous control mentioned
Zuberi et al. (27)	2015	India	RT-qPCR- customized primer	200a , 200b, 200c	Serum	Serous, Mucinous, Clear cell, Endometrioid	70 EOC, 70 Healthy	snU6
Gao et al. (7)	2015	China	RT-qPCR-TaqMan miRNA reverse transcription kit (Applied Biosystems)	200c , 141	Serum	Serous, Mucinous, Clear cell, Endometrioid	74 EOC, 19 Borderline, 50 healthy	No endogenous control mentioned

Table S2. Clinical characteristics of the ovarian cancer patients in this study

Institution	Code	Age	Histology	Stage	Grade	Metastasis (Yes/No)
BWH	BWH01	70	Serous	IV	High	Yes
BWH	BWH02	58	Serous	IIIC	High	Yes
BWH	BWH03	57	Serous	IVA	High	Yes
BWH	BWH04	57	Serous	IIIC	High	Yes
BWH	BWH05	62	Serous	IIIC	High	Yes
BWH	BWH06	54	Serous		High	Yes
BWH	BWH07	76	Serous		High	Yes
BWH	BWH08	75	Serous			
BWH	BWH09	54	Serous		High	Yes
BWH	BWH10	75	Serous	IV	High	Yes
BWH	BWH11	78	Serous	IV	High	Yes
BWH	BWH12	70	Serous	III	High	Yes
BWH	BWH13	69	Serous	IIIb	High	Yes
BWH	BWH14	66	Serous		High	
BWH	BWH15	79	Serous	II	High	No
BWH	BWH16	55	Serous		High	
BWH	BWH17	62	Serous	IIIC	High	Yes
BWH	BWH18	68	Serous	IIIC	High	Yes
BWH	BWH19	64	Serous	IV	High	Yes
BWH	BWH20	71	Serous	IIIC	High	Yes
BWH	BWH21	67	Serous	II	High	No
BWH	BWH22	74	Serous	IIIb	High	Yes
BWH	BWH23	43	Endometrioid	IC	Low	No
BWH	BWH24	77	Endometrioid		Low	No
MDA	MD1	82	Serous	IIIC	High	Yes
MDA	MD4	66	Serous	IIIC	High	Yes
MDA	MD6	71	Serous	IC	Low	No
MDA	MD7	70	Serous	IIA	High	No
MDA	MD9	69	Serous	IIIC	High	Yes
MDA	MD10	57	Serous	IIIC	High	Yes
MDA	MD12	63	Serous	IIIC	High	Yes
MDA	MD13	77	Serous	IIIC	High	yes
MDA	MD15	57	Serous	IIIC	High	yes
MDA	MD16	60	Serous	IIIC	High	yes
MDA	MD17	61	Serous	IIB	High	No
MDA	MD18	83	Serous	IIIC	High	yes
MDA	MD19	75	Serous	IIIC	High	yes
MDA	MD20	61	Serous	IIIC	High	yes
MDA	MD21	61	Serous	IIA	High	No
MDA	MD22	77	Serous	IIIC	High	Yes
MDA	MD23	71	Serous	IIIC	High	Yes
MDA	MD24	77	Serous	IIIC	High	Yes
MDA	MD25	53	Serous	IIIC	High	yes

MDA	MD27	68	Serous	IIIC	High	Yes
MDA	MD29	50	Serous	IV	High	Yes
MDA	MD30	73	Serous	IIIC	High	Yes
MDA	MD31	38	Serous	IIIC	High	Yes
MDA	MD33	72	Serous	IIIC	High	Yes
MDA	MD34	66	Serous	IIIC	High	Yes
CUHK	HK01	55	Serous	IC	High	No
CUHK	HK02	50	Serous	IC	High	No
CUHK	HK03	59	Serous	IC	High	No
CUHK	HK04	74	Serous	IIB	High	No
CUHK	HK05	56	Serous	IIC	High	No
CUHK	HK06	47	Serous	IIIB	High	Yes
CUHK	HK07	54	Serous	IIIB	High	Yes
CUHK	HK08	37	Serous	IIIB	High	Yes
CUHK	HK09	76	Serous	IIIC	High	Yes
CUHK	HK10	44	Serous	IIIC	High	Yes
CUHK	HK11	53	Serous	IIIC	High	Yes
CUHK	HK12	54	Serous	IIIC	High	Yes
CUHK	HK13	42	Serous	IIIC	High	Yes
CUHK	HK14	41	Serous	IIIC	High	Yes
CUHK	HK15	45	Serous	IIIC	High	Yes
CUHK	HK16	66	Serous	IIIC	High	Yes
CUHK	HK17	41	Serous	IIIC	High	Yes
CUHK	HK18	44	Serous	IIIC	High	Yes
CUHK	HK19	42	Serous	IIIC	High	Yes
CUHK	HK20	76	Serous	IIIC	High	Yes
CUHK	HK21	71	Serous	IV	High	Yes
CUHK	HK22	62	Clear Cell	IA	High	No
CUHK	HK23	53	Clear Cell	IA	High	No
CUHK	HK24	44	Clear Cell	IA	High	No
CUHK	HK25	53	Clear Cell	IA	High	No
CUHK	HK26	53	Clear Cell	IA		No
CUHK	HK27	52	Clear Cell	IB	High	No
CUHK	HK28	44	Clear Cell	IC	High	No
CUHK	HK29	49	Clear Cell	IC	High	No
CUHK	HK30	52	Clear Cell	IC	High	No
CUHK	HK31	43	Clear Cell	IC	High	No
CUHK	HK32	55	Clear Cell	IC	High	No
CUHK	HK33	55	Clear Cell	IC	High	No
CUHK	HK34	51	Clear Cell	IC		No

CUHK	HK35	51	Clear Cell	IC		No
CUHK	HK36	48	Clear Cell	IIB	Low	No
CUHK	HK37	47	Clear Cell	IIIC	High	Yes
CUHK	HK38	46	Clear Cell	IV	High	Yes
CUHK	HK39	63	Endometrioid	IA	Low	No
CUHK	HK40	47	Endometrioid	IC	Low	No
CUHK	HK41	62	Endometrioid	IC	Low	No
CUHK	HK42	52	Endometrioid	IC	Low	No
CUHK	HK43	46	Endometrioid	IC	Low	No
CUHK	HK44	43	Endometrioid	IC	Low	No
CUHK	HK45	46	Endometrioid	IC	Low	No
CUHK	HK46	46	Endometrioid	IC	High	No
CUHK	HK47	45	Endometrioid	IC	High	No
CUHK	HK48	39	Endometrioid	IC	High	No
CUHK	HK49	54	Endometrioid	IC	High	No
CUHK	HK50	51	Endometrioid	IIC	Low	No
CUHK	HK51	51	Endometrioid	IIC	Low	No
CUHK	HK52	30	Endometrioid	IIIC	High	Yes
CUHK	HK53	42	Endometrioid	IIIC	High	Yes
CUHK	HK54	43	Endometrioid	IIIC	High	Yes
CUHK	HK55	39	Mucinous	IA	Low	No
CUHK	HK56	48	Mucinous	IA	High	No
CUHK	HK57	67	Mucinous	IA	High	No
CUHK	HK58	38	Mucinous	IA	High	No
CUHK	HK59	65	Mucinous	IA	High	No
CUHK	HK60	64	Mucinous	IC	Low	No
CUHK	HK61	36	Mucinous	IC	High	No
CUHK	HK62	53	Mucinous	IC	High	No
CUHK	HK63	73	Mucinous	IC	High	No
CUHK	HK64	42	Mucinous	IC	High	No
CUHK	HK65	40	Mucinous	IC	High	No
CUHK	HK66	54	Mucinous	IC	High	No
CUHK	HK66	59	Mucinous	IC		No
CUHK	HK67	48	Mucinous	IIIC		Yes
CUHK	HK68	65	Mucinous	IV	High	Yes

Institutions: BWH = Brigham and Women's Hospital; MDA = MD Anderson Cancer Center;
CUHK = The Chinese University of Hong Kong

Table S3. Mean (SD) levels of miR-200 family members between Chinese and American samples

Normal	Chinese	American	*P*-value^
miR-200a	-16.8 (7.6)	-22.5 (8.7)	0.001
miR-200b	-19.4 (7.9)	-23.4 (5.9)	0.012
miR-200c	-12.3 (6.2)	-21.5 (7.2)	<0.001
miR-429	-18.0 (5.5)	-15.2 (9.2)	0.072
miR-141	-10.2 (5.4)	-21.7 (7.9)	<0.001
Cancer	Chinese	American	p-value^
miR-200a	-16.0 (9.3)	-18.8 (8.4)	0.104
miR-200b	-13.4 (10.1)	-17.5 (9.7)	0.03
miR-200c	-10.5 (10.3)	-15.6 (9.1)	0.006
miR-429	-13.6 (9.0)	-18.0 (8.7)	0.01
miR-141	-14.5 (8.6)	-16.4 (9.7)	0.256

^ *P*-value using independent-sample t-test

Table S4. Correlation analysis of miR-200 family members in all 214 samples.

All samples	miR-200a	miR-200b	miR-200c	miR-429	miR-141
miR-200a		0.377* (<0.001)	0.454* (<0.001)	0.261* (<0.001)	0.324* (<0.001)
miR-200b	0.377* (<0.001)		0.517* (<0.001)	0.225* (0.001)	0.394* (<0.001)
miR-200c	0.454* (<0.001)	0.517* (<0.001)		0.225* (0.001)	0.445* (<0.001)
miR-429	0.261* (<0.001)	0.225* (0.001)	0.225* (0.001)		0.032 (0.641)
miR-141	0.324* (<0.001)	0.394* (<0.001)	0.445* (<0.001)	0.032 (0.641)	

Data are presented as Pearson correlation coefficients (*P*-values in brackets).
* Indicates significance.

Table S5. Correlation analysis of miR-200 family members in all cancer samples (N=118).

All cancer samples	miR-200a	miR-200b	miR-200c	miR-429	miR-141
miR-200a		0.436* (<0.001)	0.532* (<0.001)	0.454* (<0.001)	0.384* (<0.001)
miR-200b	0.436* (<0.001)		0.559* (<0.001)	0.384* (<0.001)	0.462* (<0.001)
miR-200c	0.532* (<0.001)	0.559* (<0.001)		0.469* (<0.001)	0.423* (<0.001)
miR-429	0.454* (<0.001)	0.384* (<0.001)	0.469* (<0.001)		0.172 (0.062)
miR-141	0.384* (<0.001)	0.462* (<0.001)	0.423* (<0.001)	0.172 (0.062)	

Data are presented as Pearson correlation coefficients (*P*-values in brackets).
* Indicates significance.

Table S6. Correlation analysis of miR-200 family members in all normal samples (N=96).

All normal samples	miR-200a	miR-200b	miR-200c	miR-429	miR-141
miR-200a		0.249* (0.014)	0.306* (0.002)	-0.079 (0.445)	0.256* (0.012)
miR-200b	0.249* (0.014)		0.364* (<0.001)	-0.165 (0.108)	0.382* (<0.001)
miR-200c	0.306* (0.002)	0.364* (<0.001)		-0.297* (0.003)	0.526* (<0.001)
miR-429	-0.079 (0.445)	-0.165 (0.108)	-0.297* (0.003)		-0.190 (0.064)
miR-141	0.256* (0.012)	0.382* (<0.001)	0.526* (<0.001)	-0.190 (0.064)	

Data are presented as Pearson correlation coefficients (*P*-values in brackets).
* Indicates significance.

Table S7. Correlation analysis of miR-200 family members in all American samples (N=84).

American samples	miR-200a	miR-200b	miR-200c	miR-429	miR-141
miR-200a		0.137 (0.213)	0.399* (<0.001)	0.027 (0.807)	0.295* (0.006)
miR-200b	0.137 (0.213)		0.454* (<0.001)	-0.033 (0.763)	0.416* (<0.001)
miR-200c	0.399* (<0.001)	0.454* (<0.001)		-0.036 (0.742)	0.391* (<0.001)
miR-429	0.027 (0.807)	-0.033 (0.763)	-0.036 (0.742)		-0.108 (0.328)
miR-141	0.295* (0.006)	0.416* (<0.001)	0.391* (<0.001)	-0.108 (0.328)	

Data are presented as Pearson correlation coefficients (*P*-values in brackets).
* Indicates significance.

Table S8. Correlation analysis of miR-200 family members in American cancer samples (N=49).

American cancer samples	miR-200a	miR-200b	miR-200c	miR-429	miR-141
miR-200a		0.178 (0.222)	0.414* (0.003)	0.256 (0.075)	0.465* (0.001)
miR-200b	0.178 (0.222)		0.385* (0.006)	0.243 (0.092)	0.364* (0.010)
miR-200c	0.414* (0.003)	0.385* (0.006)		0.219 (0.131)	0.370* (0.009)
miR-429	0.256 (0.075)	0.243 (0.092)	0.219 (0.131)		0.065 (0.658)
miR-141	0.465* (0.001)	0.364* (0.010)	0.370* (0.009)	0.065 (0.658)	

Data are presented as Pearson correlation coefficients (*P*-values in brackets).
* Indicates significance.

Table S9. Correlation analysis of miR-200 family members in American normal samples (N=35).

American normal samples	miR-200a	miR-200b	miR-200c	miR-429	miR-141
miR-200a		-0.158 (0.364)	0.262 (0.129)	-0.188 (0.279)	-0.102 (0.560)
miR-200b	-0.158 (0.364)		0.396* (0.019)	-0.465* (0.005)	0.347* (0.041)
miR-200c	0.262 (0.129)	0.396* (0.019)		-0.323 (0.058)	0.238 (0.168)
miR-429	-0.188 (0.279)	-0.465* (0.005)	-0.323 (0.058)		-0.285 (0.097)
miR-141	-0.102 (0.560)	0.347* (0.041)	0.238 (0.168)	-0.285 (0.097)	

Data are presented as Pearson correlation coefficients (*P*-values in brackets).
* Indicates significance.

Table S10. Correlation analysis of miR-200 family members in all Chinese samples (N=130).

Chinese samples	miR-200a	miR-200b	miR-200c	miR-429	miR-141
miR-200a		0.478* (<0.001)	0.422* (<0.001)	0.422* (<0.001)	0.255* (0.003)
miR-200b	0.478* (<0.001)		0.511* (<0.001)	0.385* (<0.001)	0.320* (<0.001)
miR-200c	0.422* (<0.001)	0.511* (<0.001)		0.403* (<0.001)	0.353* (<0.001)
miR-429	0.422* (<0.001)	0.385* (<0.001)	0.403* (<0.001)		0.115 (0.194)
miR-141	0.255* (0.003)	0.320* (<0.001)	0.353* (<0.001)	0.115 (0.194)	

Data are presented as Pearson correlation coefficients (*P*-values in brackets).
* Indicates significance.

Table S11. Correlation analysis of miR-200 family members in Chinese cancer samples (N=69).

Chinese cancer samples	miR-200a	miR-200b	miR-200c	miR-429	miR-141
miR-200a		0.566* (<0.001)	0.575* (<0.001)	0.547* (<0.001)	0.313* (0.009)
miR-200b	0.566* (<0.001)		0.628* (<0.001)	0.425* (<0.001)	0.523* (<0.001)
miR-200c	0.575* (<0.001)	0.628* (<0.001)		0.566* (<0.001)	0.448* (<0.001)
miR-429	0.547* (<0.001)	0.425* (<0.001)	0.566* (<0.001)		0.221 (0.068)
miR-141	0.313* (0.009)	0.523* (<0.001)	0.448* (<0.001)	0.221 (0.068)	

Data are presented as Pearson correlation coefficients (*P*-values in brackets).
* Indicates significance.

Table S12. Correlation analysis of miR-200 family members in Chinese normal samples (N=61).

Chinese normal samples	miR-200a	miR-200b	miR-200c	miR-429	miR-141
miR-200a		0.351* (0.006)	0.078 (0.548)	0.163 (0.210)	0.214 (0.098)
miR-200b	0.351* (0.006)		0.225 (0.082)	0.103 (0.431)	0.286* (0.025)
miR-200c	0.078 (0.548)	0.225 (0.082)		-0.153 (0.239)	0.269* (0.036)
miR-429	0.163 (0.210)	0.103 (0.431)	-0.153 (0.239)		0.179 (0.168)
miR-141	0.214 (0.098)	0.286* (0.025)	0.269* (0.036)	0.179 (0.168)	

Data are presented as Pearson correlation coefficients (*P*-values in brackets).
* Indicates significance.

Table S13. Correlation analysis of miR-200 family members in laying hens (N=27).

All chicken samples	miR-200a	miR-200b	miR-429
miR-200a		0.817* (<0.001)	0.535* (0.005)
miR-200b	0.817* (<0.001)		0.661* (<0.001)
miR-429	0.535* (0.005)	0.661* (<0.001)	

Data are presented as Pearson correlation coefficients (*P*-values in brackets).
* Indicates significance.
